# Pharmacological and non-pharmacological interventions for osteoporosis: A protocol for an overview with an evidence map and a network meta-analysis of trials

**DOI:** 10.1097/MD.0000000000026429

**Published:** 2021-06-18

**Authors:** Jidong Tian, Shuo Wu, Lin Dong, Hao Tang

**Affiliations:** a Gansu Provincial Hospital of Traditional Chinese Medicine; b The First People's Hospital of Jiayuguan City, Jiayuguan, Gansu; c Clinical College of Chinese Medicine, Gansu University of Chinese Medicine, Lanzhou, China.

**Keywords:** A MeaSurement Tool to Assess systematic Reviews 2, Grading of Recommendations Assessment, Development and Evaluation, network meta-analysis, osteoporosis, overview

## Abstract

**Background:**

Osteoporosis is a common bone disease that has a significant social and economic effect. Many meta-analyses of pharmacological and non-pharmacological treatments for osteoporosis have been reported, but the findings may be contradictory, and both the reporting and methodological quality remain unknown. As a result, an overview that includes a network meta-analysis was proposed to address these issues.

**Methods:**

The Cochrane library, PubMed, Embase, CBM, and CNKI databases will be systematically searched for meta-analyses of osteoporosis interventions from inception to May 2021. In order to evaluate the reporting and methodological quality of each included meta-analysis, Preferred Reporting Items for Systematic Review and Meta-analysis 2020 (PRISMA-2020), and A MeaSurement Tool to Assess systematic Reviews 2 (AMSTAR-2) will be used. For the assessment of the relative efficacy and safety of treatments reported in the randomized controlled trials included in the meta-analyses identified by the overview, a Bayesian network meta-analysis will be carried out. The odds ratio and standard mean difference with their 95% credible intervals will be used to present the binary and continuous outcomes, respectively, and the Grading of Recommendations Assessment, Development and Evaluation method will be used to determine the certainty of the evidence through Confidence In Network Meta-Analysis. Data analysis will be performed using WinBUGS, R, and Stata, with a 2-sided *P *< .05 considered as statistically significant.

**Results:**

The findings of this overview, which includes a network meta-analysis, will be submitted to a peer-reviewed journal for publication.

**Conclusion:**

An overview with network meta-analysis will provide evidence on the efficacy and safety of pharmacological and non-pharmacological interventions for osteoporosis, while also identifying the flaws in previously published meta-analyses. All of these results may be used to improve clinical decision-making and future studies.

**INPLASY registration number:**

INPLASY202150022.

## 1 Introduction

Osteoporosis is a common skeletal condition that is characterized by loss of bone mass and fragility fracture.^[1–3]^ Globally, osteoporosis and related fractures often severely affect the health and quality of life of sufferers^[2]^ and have placed a massive burden on the economy.^[3]^ According to 2010 estimates, the overall prevalence of osteoporosis in communities in the United States was 10.3%,^[2]^ while there was a reported 3.5 million cases of osteoporotic fractures in the European Union.^[3]^ The number of osteoporotic fracture cases is projected to reach 4.5 million a year by 2025^[3]^ and the direct treatment of osteoporotic fractures costs between 5000 and 6500 billion dollars per year in Canada, Europe, and the USA alone, based on a systematic review published in 2020.^[3]^ At present, western medicines (e.g., bisphosphonates, teriparatide, and calcitonin),^[4–6]^ traditional Chinese medicine,^[7]^ traditional Chinese exercises (e.g., Wuqinxi and Taijiquan),^[8,9]^ acupuncture therapy,^[10]^ and dietary supplements (e.g., calcium and vitamin D)^[11]^ are commonly used as interventions for osteoporosis. Meta-analyses are considered as the highest level of evidence in the era of evidence-based medicine,^[12]^ with many of these reviews on the interventions for osteoporosis published in peer-reviewed journals.^[5–8,10,11]^ For example, in a meta-analysis published by Yuan et al^[6]^ in 2019, the results of a comparison between teriparatide and bisphosphonates for the treatment of postmenopausal osteoporosis were reported. Furthermore, Pan et al^[10]^ published a systematic review with a meta-analysis in 2018 that evaluated the effectiveness of acupuncture for osteoporosis.

An overview of systematic reviews is a commonly used method to summarize the results of published meta-analyses in a specific healthcare field,^[12–14]^ and it has been widely used in various areas, such as rheumatoid arthritis,^[15]^ snakebite envenoming,^[16]^ and appendicitis.^[17]^ A network meta-analysis is a tool for comparing and pooling evidence from multiple interventions,^[18,19]^ as well as providing a relative ranking of these interventions in terms of clinical outcome.^[18,19]^ Despite the fact that many systematic reviews with meta-analyses of osteoporosis interventions have been published, their results may be inconsistent or even conflicting. Furthermore, the reporting and methodological quality of these meta-analyses are unknown, and these may affect the clinical practicability and scientific reliability of the results.^[20]^ Therefore, we designed an overview to evaluate both the reporting and methodological quality of meta-analyses of osteoporosis interventions. In addition, a network meta-analysis will be carried out to compare the relative efficacy and safety of all pharmacological and non-pharmacological treatments for osteoporosis that were reported in randomized controlled trials included in the meta-analyses identified by this overview.

## 2 Methods

The present overview with network meta-analysis has been registered on the International Platform of Registered Systematic Review and Meta-analysis Protocols (registration number: INPLASY202150022). The current study was prepared according to the Preferred Reporting Items for Systematic Review and Meta-analysis Protocols checklist (Supplementary file).^[21]^

### 2.1 Eligibility criteria

#### 2.1.1 Type of study

All systematic reviews with meta-analyses of osteoporosis interventions that have been published in English or Chinese peer-reviewed journals will be included in the overview section. For the network meta-analysis section, randomized controlled trials that were identified from the systematic reviews with meta-analyses in the overview section will be included.

#### 2.1.2 Type of participant

Patients that were diagnosed with osteoporosis using World Health Organization criteria^[1]^ or other commonly used standards will be included in the present overview with network meta-analysis. No restrictions will be placed on the sex, age, ethnicity, or race of the patients.

#### 2.1.3 Type of intervention

The intervention group received pharmacological, non-pharmacological treatments, or a combination of both, including but not limited to, western drugs (e.g., bisphosphonates, calcitonin, and teriparatide), dietary supplements (e.g., calcium and vitamin D), acupuncture, traditional Chinese medicine, and traditional Chinese exercises (e.g., Wuqinxi and Taijiquan). The control group was either given a placebo, no treatment, or any available active treatments. There are no restrictions on the usage, dosage, and duration of treatment for both groups.

#### 2.1.4 Type of outcome

In this overview with network meta-analysis, primary outcomes will include the effective rate, bone mineral density, and pain improvement, while secondary outcomes will include the level of serum calcium, quality of life, and any adverse events.

### 2.2 Exclusion criteria

Qualitative systematic reviews, narrative reviews, basic experimental studies, protocols, conference abstracts, cohort studies, cross-sectional studies, case-control studies, preclinical studies, and any articles that do not include full-texts or required data will be excluded.

### 2.3 Search methods

The PubMed, Cochrane library, Embase, CBM, and CNKI databases will be systematically searched from inception to May 2021 for published systematic reviews with meta-analyses of interventions for osteoporosis. In addition, the reference lists of the included systematic reviews with meta-analyses will be examined to identify any other potentially relevant articles. The details of the search strategy using PubMed are presented in Table 1.

**Table 1:** The search strategy of PubMed database.

Search strategy	
#1	“Osteoporosis” [Mesh] OR “Osteoporosis, Postmenopausal” [Mesh] OR osteoporosis [Title/Abstract] OR osteoporosis [Title/Abstract] OR “bone loss” [Title/Abstract] OR “bone losses” [Title/Abstract]
#2	“Systematic Review” [Publication Type] OR “Systematic Reviews as topic” [Mesh] OR “Network Meta-Analysis” [Mesh] OR “Meta-analysis” [Publication Type] OR “Meta-analysis as topic” [Mesh] OR “systematic review” [Title/Abstract] OR “meta-analysis” [Title/Abstract]
#3	#1 AND #2

### 2.4 Study screening and data extraction

**Figure 1:**
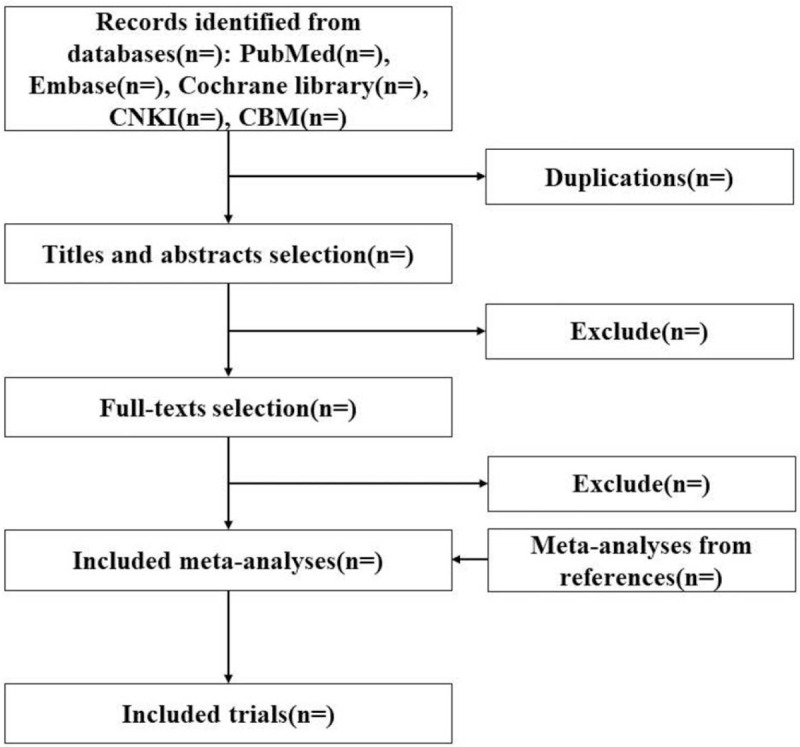
The selection flowchart of this overview with network meta-analysis.

Two reviewers will independently screen records and extract data from systematic reviews and randomized controlled trials that were included in this overview. Any arising conflicts will be resolved through discussion or consultation. All identified records from the databases will be imported into Endnote (Version X9, Clarivate Analytics, USA) for the removal of duplicated records and further screening. Firstly, the titles and abstracts will be screened. Secondly, the full texts of potentially relevant publications will be downloaded in order to identify relevant systematic reviews with meta-analyses. Thirdly, the reference lists of relevant meta-analyses will be screened to identify relevant randomized controlled trials of pharmacological or non-pharmacological interventions for osteoporosis. The selection flowchart of this overview with network meta-analysis is shown in Fig. 1.

The following information of each included meta-analysis will be extracted: title, first author, year of publication, country of the corresponding author, journal, types of osteoporosis, interventions and comparators, number of included primary studies, number of randomized controlled trials, total sample size of each meta-analysis, and funding. For each identified trial, the following data will be extracted: title, first author, year of publication, setting, outcomes of interest, interventions and comparators, duration of treatment, anatomic sites of osteoporosis, number of total cases, number of patients, sex, age, types of osteoporosis (e.g., postmenopausal osteoporosis, senile osteoporosis, secondary osteoporosis), and sources of funding.

### 2.5 Assessment of evidence quality

#### 2.5.1 The quality of systematic reviews

The A MeaSurement Tool to Assess systematic Reviews 2 (AMSTAR-2)^[22]^ and Preferred Reporting Items for Systematic Review and Meta-analysis 2020 (PRISMA-2020)^[23]^ will be used by 2 independent reviewers to assess the methodological and reporting quality of each included meta-analysis, respectively. In order to answer the questions on the methodological and reporting quality for the AMSTAR-2 and PRISMA-2020 used in the overview, 3 possible responses are available: “Yes,” “No,” or “Partial yes.” In addition, the overall methodological quality of each systematic review will be rated: high, moderate, low, or critically low. Any disputes will be resolved by consensus.

#### 2.5.2 Risk of bias in randomized controlled trials

The risk of bias in randomized controlled trials will be evaluated using the Cochrane risk of bias tool,^[24]^ which includes 7 aspects: random sequence generation, allocation concealment, blinding of participants and personnel, blinding of outcome assessment, incomplete outcome data, selective reporting, and other bias. Each aspect may be classified as having a low, unclear, or high risk of bias.^[24]^ A trial is considered low quality if the random sequence generation, allocation concealment, or blinding were evaluated as having a high risk of bias, regardless of the risk in other domains; otherwise, it is considered high quality.^[25]^

#### 2.5.3 Certainty of the evidence

The Grading of Recommendations Assessment, Development and Evaluation^[26]^ method will be used to assess the certainty of the evidence for primary outcomes include effective rate, bone mineral density, and pain improvement. The online application “Confidence In Network Meta-Analysis”^[26]^ will be used to complete this process and it includes 6 domains: within-study bias, reporting bias, indirectness, imprecision, heterogeneity, and incoherence. Finally, each outcome would be rated: high, moderate, low, or very low.^[26]^

### 2.6 Statistical analysis

The results of the included systematic reviews’ methodological and reporting quality will be presented as a number and a percentage, and the evidence mapping method^[27]^ will be used to visualize the results. The Bayesian network meta-analysis will be performed using the Markov Chain Monte-Carlo, and statistical analyses will be conducted using the WinBUGS 1.4.3 (MRC Biostatistics Unit, Cambridge, UK) and the *gemtc* package in R 4.0.2 (R Core Team, Vienna, Austria). Following an initial burn-in of 20 000, three Markov chains with 100,000 iterations will be included in the analyses. The inconsistency between direct and indirect estimates will be checked using the node splitting method.^[28]^ The relative efficacy and safety of different interventions will be ranked using surface under the cumulative ranking area.^[29]^ Generation of the network plots of network meta-analyses as well as conventional meta-analyses will be performed using the Stata 16.0 (StataCorp, College Station, TX) software. The statistical heterogeneity across trials will be assessed using *I*^2^ statistics; significant heterogeneity is defined when *I*^2^ > 50%. The effect sizes of binary and continuous outcomes will be presented using the standard mean difference and odds ratio with 95% credible intervals, respectively. Subgroup analyses will be conducted for the conventional meta-analyses based on several variables including the age and sex of patients, anatomic sites of osteoporosis, types of osteoporosis, duration of treatment, and quality of trials. A sensitivity analysis will be performed by excluding trials with small sample sizes (N < 60). The funnel plots and Egger test will be used to determine publication bias. A 2-sided *P* < 0.05 will be considered statistically significant.

## 3 Discussion

Osteoporosis is a common bone disease that places a significant social and economic burden, often resulting in fragility fractures due to bone mass loss.^[1–3]^ Therefore, it is important to carry out early prevention, diagnosis, and effective treatment of osteoporosis. Although a large number of systematic reviews with meta-analyses of pharmacological and non-pharmacological treatments for osteoporosis have been published in peer-reviewed journals,^[5–8,10,11]^ their reporting and methodological quality is unclear. In addition, the results that have been reported may be contradictory. Hence, the findings of this present study may address the aforementioned issues through the overview and network meta-analysis methods.

This overview with network meta-analysis has several strengths. Firstly, this is the first overview to include published meta-analyses of pharmacological and non-pharmacological treatments for osteoporosis. Furthermore, the reporting and methodological quality of identified meta-analyses can be determined using the PRISMA-2020 and AMSTAR-2 tools, and an evidence mapping method will be used to display the assessment results. Secondly, the Bayesian network meta-analysis that will be conducted is able to pool and compare all available treatments that were reported in trials included in the meta-analyses identified by the overview. The ranking results of these interventions can be used to aid the establishment of clinical practice guidelines for clinical decision-making.^[30]^ This study, however, also has several limitations. Firstly, considering our language background, meta-analyses alone published in English or Chinese journals will be included. Secondly, rather than re-searching for new trials, a Bayesian network meta-analysis will be conducted based on trials identified in the overview but this method has previously been employed in other fields, for example, irritable bowel syndrome^[31]^ and allergic rhinitis.^[32]^

## 4 Ethics and dissemination

As this study does not involve any animal or human data, ethical approval is not required. The results of this study will be reported according to PRISMA extension statement for network meta-analyses,^[33]^ and then submitted to a peer-reviewed journal for publication.

## Author contributions

**Conceptualization:** Jidong Tian, Shuo Wu.

**Data curation:** Lin Dong, Hao Tang.

**Formal analysis:** Lin Dong, Hao Tang.

**Methodology:** Jidong Tian, Shuo Wu.

**Writing – original draft:** Jidong Tian.

**Writing – review & editing:** Shuo Wu, Lin Dong, Hao Tang.

## Supplementary Material

Supplemental Digital Content
